# Effect of Maltodextrin on the Physicochemical Properties and Cooking Performance of Sweet Potato Starch Noodles

**DOI:** 10.3390/foods11244082

**Published:** 2022-12-16

**Authors:** Weiwei Hu, Wen Zhang, Zhiguo Zhang, Shengfa Shen, Guoquan Lu, Weicheng Wu

**Affiliations:** 1 State Key Laboratory for Managing Biotic and Chemical Threats to the Quality and Safety of Agro-products, Food Science Institute, Zhejiang Academy of Agricultural Sciences, Hangzhou 310021, China; 2 College of Food and Pharmacy, Zhejiang Ocean University, Zhoushan 316022, China; 3 Institute of Crops and Nuclear Technology Utilization, Zhejiang Academy of Agricultural Sciences, Hangzhou 310021, China; 4 The Key Laboratory for Quality Improvement of Agricultural Products of Zhejiang Province, College of Advanced Agricultural Sciences, Zhejiang A&F University, Hangzhou 311300, China

**Keywords:** sweet potato starch noodle, maltodextrin, physicochemical properties, cooking quality, interaction

## Abstract

Maltodextrin (MD), the hydrolyzed starch product, is a promising alternative ingredient to improve the quality of starch-based foods. The effects of MD on the physicochemical, microstructural, and cooking properties of sweet potato starch (SPS) noodles, as well as the mechanism of SPS-MD interactions, are discussed. Fourier transform infrared spectroscopy (FTIR) and X-ray diffraction (XRD) results indicated that MD at a suitable concentration can improve the ordered structure of SPS-MD gels. The cooking loss showed lower values of 1.47–2.16% at 0.5–2.0 wt% MD. For the texture properties, an increase in hardness and chewiness occurred at first with the addition of MD, followed by a decreasing trend, showing a maximum value at 2.0 wt% of MD. The pasting and thermal results verified the increased stability of the starch granules with MD < 3 wt%. Additionally, SPS formed a solid-like gel with MD, and the main interaction forces between SPS and MD were hydrogen bonding. The scanning electron microscopy results revealed that the higher concentrations of MD (>3 wt%) loosened the gel structure and markedly increased the pore size. These results help us to better understand the interaction mechanism of the SPS-MD complex and facilitate the development of SPS-based gel products.

## 1. Introduction

Starch noodles, with their transparent glossiness and fine shape, are one of the most popular staple foods consumed in Asian countries [1,2]. Unlike wheat flour-based noodles, starch noodles are only produced with purified plant starches [3]. In this regard, they are one of the foods marketed with growth projection, as gluten should be eliminated from the diet of people suffering from celiac disease [4]. Industrially, sweet potato starch (SPS) is the traditional material used in production due to its low price and high output. Starch accounts for 70% to 80% of the dry matter (DM) content of sweet potato root tuber, and sweet potatoes have the ability to grow in poor soils with little pesticides, fertilizer, and effort [5]. However, the development and utilization of natural SPS are curtailed partly due to its intrinsic properties, including the degree of swelling and poor solubility, retrogradation tendency, and strong paste viscosity [6]. For example, the high viscosity of SPS pastes commonly induces poor transparency and hardness of the resulting products, thereby reducing consumer acceptance. On the other hand, the texture is one of the primary quality parameters for starch noodles. Excellent starch noodles should have a low cooking loss and high tensile strength within the cooking time [7,8]. Nevertheless, the absence of proteins forming the gluten network makes it difficult to form a cohesive and uniform matrix.

In fact, starch noodles are a form of dried starch gels. Starch is mostly gelatinized during cooking, and the semicrystalline structure is disrupted and transforms into an amorphous state. In the cooling stage, starch molecules undergo a disorder-to-order transition via starch retrogradation, and eventually connect to form an interconnected three-dimensional gel network [9]. Thus, in order to enhance the cooking performance of noodles, many investigators have attempted the modification of starches to tune their physiochemical properties and associated quality attributes of the related products. The modification methods can be divided into physical, chemical, and enzymatic processes [10,11]. Physically mixing starch with food additives is a simple, effective, and environmentally friendly method that is widely investigated by researchers. Moreover, the addition of polysaccharides is more attractive compared to glycerol monostearate and fatty-acid esters, as they can have specific interactions with starch (e.g., chemically or physically), the main component in starch noodles, thus presenting a specific quality on the end product [12,13]. Xanthan can stabilize the gluten-free networks by controlling the water molecule mobility and improving the intermolecular viscosity [14]. *Mesona chinensis* Benth polysaccharide (MCP) could cross-link with the SPS, enhancing the mixture gel viscoelasticity [15]. Feng et al. (2020) compared the effects of proteins (gluten, egg white protein) and polysaccharides (chitosan, xanthan, and sodium alginate) on the dough rheological and texture properties of wet sweet potato vermicelli and found that all of the additions can improve the textural and cooking properties of noodles [16]. Comparatively, polysaccharides formed a stronger starch gel network than proteins.

Recently, polymer products from the hydrolysis of polysaccharides have been regarded as another alternative approach to tuning the starch retrogradation and structural properties [17,18,19]. Typically, maltodextrin (MD), the hydrolyzed starch product with a dextrose equivalent (DE) of 2–36.5, has the potential to improve the cooking quality of noodles [20,21]. Additionally, using MD as an additive presents several advantages, such as its wide availability, low cost, reduced viscosity, and film-forming properties, and its ample compatibility with different starch materials [22,23]. However, few reports have discussed the effect of MD on SPS-based starch productions and there is limited information about the mechanism of starch-MD interactions. Therefore, this work aimed to discuss the effects of MD incorporation on the physicochemical and structural changes and the resulting contributions to the quality of starch gels products (e.g., starch noodles). Additionally, the rheological analysis was also conducted to explore the interaction between SPS and MD. These results help to better understand the gel formation mechanism of the SPS-MD complex and provide a perspective regarding the application of MD to improve the quality of starch-based food.

## 2. Materials and Methods

### 2.1. Materials and Reagents 

Sweet potato starches (SPS) were obtained from Chongqing Jintian Co., Ltd. (Chongqing, China). The starch was 89.50 ± 1.76 g/100 g dry basis and the moisture was 11.78 ± 0.12 g/100 g dry basis (AACC methods). Maltodextrin (MD, DE = 11–14) was purchased from Shandong Xiwang Sugar Industry Co., Ltd. (Shandong, China). Other chemicals and reagents used in the present study were of analytical grade. 

### 2.2. Preparation of Maltodextrin Introduced Sweet Potato Starch (SPS-MD) Noodles

In this work, sweet potato noodles were prepared according to the description of Li et al. (2019) [24]. Briefly, sweet potato starch (13.5 g, db) with various MD concentrations (0, 0.5, 1, 2, 3, and 5%, *w*/*w*) was first suspended in deionized water, and the final concentration of the starch slurry was 43.4% (*w*/*v*). The slurry was then spread evenly in a stainless-steel tray (22 cm × 14 cm) and allowed to float on the surface of boiling water (30 s). In this step, the starch slurry was fixed and lost flowability. Afterward, the tray was put into the boiled water and completely immersed for 60 s until it became completely gelatinized. After the gelatinization process, the tray was placed under a tap water bath for 30 s. Accordingly, a starch colloidal gel network formed during cooling. The fresh starch gel sheet was then aged at a low temperature of 4 °C for 14 h. After retrogradation, the transparent starch sheet was cut into strips (3.0 mm), and finally oven-dried at 40 °C for 6 h to obtain dried sweet potato noodles.

### 2.3. Fourier Transform Infrared Spectroscopy

The prepared SPS-MD starch noodles were ground and then sieved by the 150-mesh sieve. The powder (~2 mg) was mixed with potassium bromide at a ratio of 1:30. After grinding and pressing into a pellet, FTIR scanning was performed in the range of 400 to 4000 cm^−1^ at room temperature using the absorbance mode with 32 scans on the Nicolet 6700 spectrometer (Thermo Fisher Scientific, Waltham, MA, USA). Data were recorded and analyzed with the Nicolet Omnic-Atlls software (Thermo Electron Corporation, Waltham, MA, USA).

### 2.4. Powder X-ray Diffraction Measurement

The starch crystallinity of the prepared SPS-MD starch noodles was measured on an XRD Ultima IV diffractometer (Rigaku, Japan) with Cu Kα radiation [21]. The diffractogram was obtained at a step size of 0.01 and scanned over the 5° to 50° (2θ) range at a scan rate of 2°/min. The crystallinity was calculated with the Jade 5.0 software (Materials Data, Inc., Santa Ana, CA, USA).

### 2.5. Cooking Quality 

According to Xiang et al. (2018), the cooking quality of the prepared SPS-MD starch noodles, including the swelling index and cooking loss, was determined with slight modification [9]. For the dry matter content, starch gels were dried in a convective oven to a constant weight at 105 °C. Starch noodles (3 g db, *W_0_*) were bathed in boiling water for 15 min. Subsequently, the strips were removed from the cooking water, drained with filter paper (*W_1_*, g), and desiccated at 105 °C (*W_2_*, g). The swelling index (%) and cooking loss (%) were determined as follows:

(1) $$\mathrm{Swelling}\;\mathrm{index}\;(\%)=W_{1}/W_{2}\times 100$$

(2) $$\;\mathrm{Cooking}\;\mathrm{loss}\;(\%)=(W_{0}-W_{2})\;/W_{0}\times 100$$

### 2.6. Texture Profile Analysis (TPA) 

The TPA of the cooked SPS-MD starch noodles was performed with a texture analyzer TA-XT2 (Shanghai, China). The test was performed according to the method by Zhou et al. (2013) with some modifications [25]. Starch noodle samples were cooked in boiled water for 15 min, cooled rapidly in chilled water, and subjected to analysis immediately. A compression cycle test (45% compression of the original sample height) was performed twice using a spherical probe (P/0.25S) at a speed of 1 mm/s. The threshold force was set at 4.0 g and the results of hardness (g), elasticity (mm), and chewiness (g) were recorded from the arithmetic mean of ten measurements.

### 2.7. Pasting Properties 

The effect of MD on the pasting properties of sweet potato starch was evaluated by a Rapid Visco-Analyzer (RVA, TecMaster, Perten Instruments, North Ryde BC, NSW, Australia) according to Zheng et al. (2021) [26]. Sweet potato starch (1.5 g, dry basis) and an MD aqueous solution (0.5, 1, 2, 3, and 5%, *w*/*w*) were weighed to an overall value of 25 g and transferred to the aluminum cylinder of a rapid viscosity analyzer. The exudate slurries were balanced at 50 °C for 1 min, heated to 95 °C at a rate of 12 °C/min, held for 5 min, then cooled down to 50 °C in 3.75 min, and finally held for 2 min. The peak and trough viscosity, breakdown viscosity, and final and setback viscosities were recorded.

### 2.8. Thermal Properties 

The degree of starch retrogradation in the prepared SPS-MD starch noodles was measured on a differential scanning calorimeter (DSC 1, Mettler Toledo, Greifensee, Switzerland) [24]. Each starch sample (approximately 5.0 mg) was weighed into steel pans of mixed distilled water (1:3 *w*/*w* sample:water ratio). The pans were sealed and left overnight at room temperature. The pan was heated from 30 °C to 90 °C at a rate of 10 °C/min and an empty pan was used as a reference. The enthalpy of gelatinization (ΔH), gelatinization onset temperature (To), peak temperature (Tp), and conclusion temperature (Tc) were measured from the endotherm in the DSC thermograms.

### 2.9. Gel Structure Morphology

The cooked SPS-MD starch noodles were cut into 3 mm sections and freeze-dried for morphological characterization. The cross-sections were coated with gold (Au) by a HITACHIE 1020 ion sputter (JEOL, Tokyo, Japan). The microstructure of the gels was photographed by a SU8100 scanning electron microscope (Hitachi, Tokyo, Japan) at the accelerating voltage of 3.0 kV.

### 2.10. Interaction Force Test

The interaction forces between sweet potato starch and maltodextrin were analyzed according to Ren et al. (2020) [15]. Briefly, 1470 mg of SPS and 30 mg of MD (SPS-2% MD, *w*/*w*) were steeped in 25 mL of distilled water and then mixed with urea (0.2 M, 0.6 M, and 1.0 M) or sodium chloride (0.2 M, 0.6 M, and 1.0 M). The mixtures were heated at 95 °C for 2 min and then cooled to ambient temperature. 

The viscoelasticity of the SPS-MD-Urea and SPS-MD-NaCl gels was analyzed with the MCR302 rheometer (Anton Paar, Graz, Austria) and a parallel plate (No. PP50). The dynamic modulus was measured at 1% strain, which was in the linear viscoelastic region, at 25 °C over an angular frequency of 0.1–100 rad/s. The loss tangent value tan(δ) was expressed by Equation (3) and the Power-Law model was calculated as shown in Equation (4):

(3) tan(δ)=G″/ G′,

(4) G′=K(ω)n

where G′ and G″ are the storage modulus and the loss modulus, respectively. *K* is the consistency index, *n* is the flow behavior index, and *ω* is the angular frequency.

### 2.11. Statistical Analysis 

Data were expressed in means ± standard deviations. One-way analysis of variance (ANOVA) test was performed by the SPSS Statistics 20.0 (IBM, Armonk, NY, USA), and *p* < 0.05 was considered statistically significant. All figures were realized by Origin 2018 (Origin Lab Corporation, Northampton, MA, USA). 

## 3. Results and Discussion

### 3.1. FTIR Analysis 

**Figure 1:**
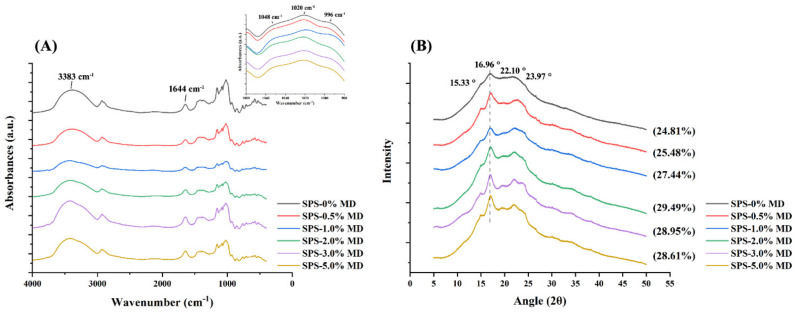
The FT−IR spectra (**A**) and XRD patterns (**B**) of sweet potato starch (SPS) noodles with various (0, 0.5, 1, 2, 3, and 5%, *w*/*w*) maltodextrin (MD) concentrations. Inset: the enlarged peaks in the range of 980−1080 cm^−1^ of each sample.

Figure 1A displayed the FT-IR spectra of sweet potato starch gels with or without maltodextrin. Compared to the SPS-0% MD, neither a new peak appeared nor absorbed peak loss in the gel samples with various MD concentrations, indicating that there were no chemical bond modifications or covalent bond formations [27]. Specifically, the 3383 cm^−1^ peak showed the O-H stretching vibration of hydroxyl groups in starch, which was attributed to intramolecular and intermolecular hydrogen bonds [28]. In this work, the slight modification in the width of the C-H band may indicate an intensified level of hydrogen bonds with the addition of MD in the SPS-MS complexes [26]. The absorption peak at 1644 cm^−1^ was associated with the stretching vibration of C=O. The inset in Figure 1A showed the enlarged peaks in the range of 980–1080 cm^−1^ of each sample. The bands at 1048 and 1020 cm^−1^ resulted from crystalline and amorphous domains of starch, respectively. The absorbance at 996 cm^−1^ corresponds to C-OH bending vibrations [29,30]. Generally, the absorbance ratio of 1048/1020 cm^−1^ was regarded as a measure to quantify the ordered degree of the external structure of starch, and the IR ratio of 1020/996 cm^−1^ can be used as an index of the proportion of the amorphous to a hydrated fraction. As shown in Table 1, the IR ratio of 1048/1020 cm^−1^ displayed a tendency of first increasing and then decreasing, with the increased proportion of MD, and the ratio of 1020/996 cm^−1^ was relatively larger when the MD concentration was higher than 3 wt%. The result implied that a low concentration of MD (<3 wt%) intensified the interactions between starch chains and contributed to a relatively ordered structure, while excessive MD may block the interactions, transforming into an amorphous phase.

### 3.2. XRD Analysis 

The crystallinity of the SPS-MD starch gels was characterized by XRD patterns and displayed in Figure 1B. All prepared samples showed a typical B-type crystal pattern with diffraction peaks at 2θ of 15.33°, 16.96°, 22.10°, and 23.97° [31]. No new peaks were observed across the spectrum upon the addition of MD, indicating that the addition of MD with amounts covered by this work had little effect on the crystalline structure of the resulting complex gels. Compared to the pure sweet potato starch gel, MD enhanced the relative crystallinity of the SPS-MD mixtures, and with the increase in the maltodextrin content, the value increased from 25.48% (SPS-0.5% MD) to 29.49% (SPS-2.0% MD). Then, the growth trend tends to be flat. The results can be interpreted as small molecules of maltodextrin connected with starch chains via hydrogen bonds when the amount of MD was lower than 3 wt%, while a large amount of MD would destroy or weaken the intra-molecular hydrogen bonds, thereby preventing the recrystallization of starch molecules. Xu et al. (2022) reported a continuous declining trend of relative crystallinity with an increased maltodextrin content [21]. The difference may be due to the V-type crystal structure formed in the whole buckwheat noodles, and MD inhibits the recrystallization of amylose-lipid combinations.

### 3.3. Analysis of Cooking and Texture Properties

The cooking properties of sweet potato noodles with different concentrations of MD are shown in Table 1. The swelling index of all tested SPS-MD combinations was comparable to that of pure sweet potato starch gel, except for the highest swelling index measured from the maximum MD concentration (*p* < 0.05). This was presumably because of the strong hydrophilicity of MD, which makes it easy for water to enter the noodles [20]. The cooking loss of SPS-MD starch noodles showed opposite trends. As shown in Table 1, with the increasing amounts of MD, the cooking loss of noodles decreased first and then increased, reaching the minimum value of 1.47 ± 0.15% as the MD was 0.5 wt% (*p* < 0.05). This can be explained as follows: on the one hand, MD cross-linked the starch fragments when a small amount of MD was applied. These starch fragments as well as MD molecules were not readily dissolved in water during cooking, therefore yielding lower cooking losses than the control. On the other hand, a higher amount of MD (>3 wt%) weakened or destroyed the intramolecular hydrogens, which was accompanied by dissolution and leaching into the boiling water [32]. This led to an increased cooking loss.

In terms of texture properties, hardness, elasticity, and chewiness are the main parameters often linked to sensory perception. As shown in Table 1, a significant increase in the hardness from 940.12 ± 37.25 g to 1583.92 ± 89.09 g (*p* < 0.05) could be obtained with an increased MD concentration (0.5–2.0 wt%). Conversely, no further enhancement was observed with a greater additive amount and even a slight decrease to 1362.22 ± 117.86 g in SPS-5.0% MD samples. Similarly, the chewiness increased to the maximum value (1160.61 ± 92.52 g, SPS-2.0% MD) and then decreased with higher MD contents. MD stabilized the internal gel network of the noodle via hydrogen bonding. However, excessive free MD would absorb water constantly and the corresponding starch noodle becomes swollen, weakening the starch gel structure [33]. Additionally, although the elasticity of starch noodles showed a decreasing trend with an increase in the MD content, the values were not significantly different.

### 3.4. RVA Pasting Profiles 

**Figure 2:**
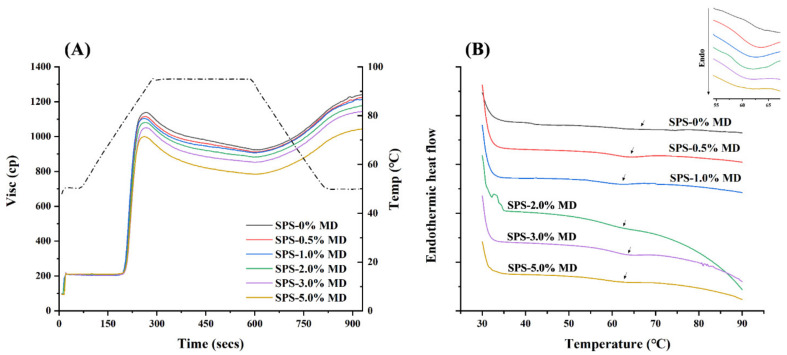
The pasting curves (**A**) and DSC patterns (**B**) of sweet potato starch (SPS) noodles with various (0, 0.5, 1, 2, 3, and 5%, *w*/*w*) maltodextrin (MD) concentrations. Inset: the enlarged endothermic peak of each sample.

The pasting curves and parameters of the SPS-MD mixture are presented in Figure 2A and Table 2, respectively. The gelatinization properties of sweet potato starch were mainly affected by the introduction of maltodextrin. SPS had the highest peak viscosity value of 1137.8 ± 11.75 cp. The peak and final viscosity notably decreased with the addition of MD. The peak viscosity reflects the ability of starch granules to swell and the competition of free water for unhydrolyzed starch granules with leached amylose [34]. In this work, MD weakened the binding capacity of starch granules to water and inhibited the swelling power of the starches, finally leading to a reduction in the viscosity. It has also been reported that certain hydrocolloids, such as pullulan [35] and xanthan [36], can reduce the hot paste viscosity by decreasing the swelling volume of starch granules during pasting. 

The breakdown viscosity (BD) value evaluates the ease of disintegration of swollen starch granules. Lower BD values indicate the larger shear and thermal stability of starch granules [11]. When the sweet potato starch was gelatinized with MD, the BD value decreased from 214.2 ± 7.47 cp to 197.2 ± 5.04 cp in a concentration-dependent manner, indicating that the SPS gel became more compact with the addition of MD (*p* < 0.05) [37]. A high concentration of MD should be avoided, as SPS showed less tolerance to the mechanical and thermal forces at 5 wt% MD (218.4 ± 5.92 cp). Additionally, the setback value (SB) evaluates the short-term retrogradation of starch, which is positively associated with the aging rate [37]. The SB showed a reducing tendency with an increased ratio of MD concentration. Specifically, lower concentrations of MD (0.5, 1.0, and 2.0 wt%) decreased the SB slightly (*p* > 0.05), while higher concentrations of MD (3.0 and 5.0 wt%) reduced the SB value significantly (*p* < 0.05). These results indicated that high concentrations of MD can retard sweet potato starch retrogradation. In the cooling stage, excessive MD may interact with the hydroxyl groups of starch chains to hinder the retrogradation process.

### 3.5. Thermal Properties 

Figure 2B demonstrated the DSC patterns of SPS-MD starch gels and Table 3 showed the results of enthalpy (ΔH) and onset (To), peak (Tp), and conclusion gelatinization (Tc) temperatures. The gelation temperature is related to the melting of the starch crystal structure. As shown in Table 3, a discernible decrease in Tp (64.31–60.42 °C) and Tc (70.62–64.56 °C) was observed in the complexes, which was positively associated with the MD binding. However, in the RVA measurement, no significant correlation was observed between the MD content and the gelatinization temperature of SPS. The difference might be ascribed to the test conditions, as the aluminum pan was completely sealed in the DSC analysis, which avoided the major external environmental factor [38]. The ΔH represents the amount of energy required for disrupting the double helix and ordered crystal structure [39]. The inset in Figure 2B showed the enlarged endothermic peak of each sample. The ΔH values of SPS-MD gels increased and then decreased with the increasing binding proportion of MD, but all were higher than that of pure sweet potato starch gels (2.55 J/g, SPS-0% MD). The results indicated that the binding of MD promoted more crystal structures in the sweet potato starch. The side chain of starches formed hydrogen bonds with low concentrations of MD, which stabilized the broken starch fragment and severed the high-energy structure [40]. Additionally, the stabilizing effect of 2 wt% MD on the structure of SPS-MD gels was more significant, which was in line with the XRD measurement. 

### 3.6. SEM Analysis 

**Figure 3:**
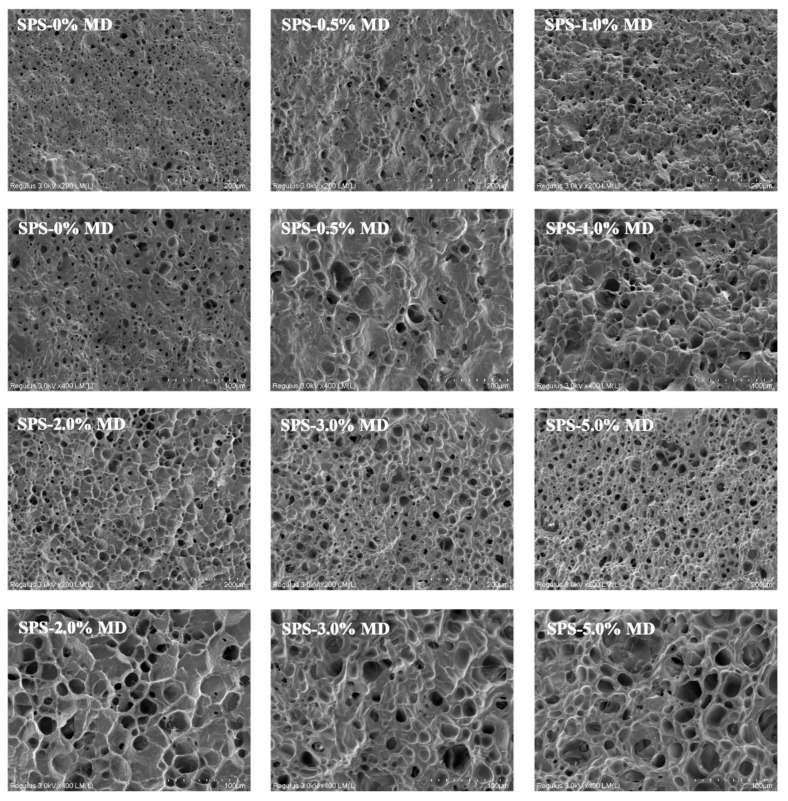
The SEM images of cooked sweet potato starch (SPS) noodles with various (0, 0.5, 1, 2, 3, and 5%, *w*/*w*) maltodextrin (MD) concentrations.

Figure 3 shows the SEM micrographs of SPS-MD starch noodles after cooking. The cross-section images showed that the prepared noodles formed pores and the pores revealed small to large sizes along with an increased MD concentration. Especially in the higher concentrations of MD (3 wt% and 5 wt%), the pore size markedly increased, and the cells were orderly and tightly connected to one another. This might be because of the higher amount of MD dissolved into water, leading to increasing pore size. In addition, the cooking loss and the sublimation of water during the lyophilization process in the preparation of SEM samples were also responsible for the formation of pore structures [9]. Additionally, it is important to note that all prepared SPS-MD gels retained their good quality and did not exhibit a collapsing behavior after adding MD. However, a larger pore size will absorb water constantly and the starch noodle will become swollen, weakening the starch gel structures, which was consistent with the conclusion of the cooking properties. Li et al. (2018) also reported that part of the porous structures would be collapsed when excess MD is incorporated in extruded instant wheat noodles [41].

### 3.7. Interactions between Sweet Potato Starch and Maltodextrin

**Figure 4:**
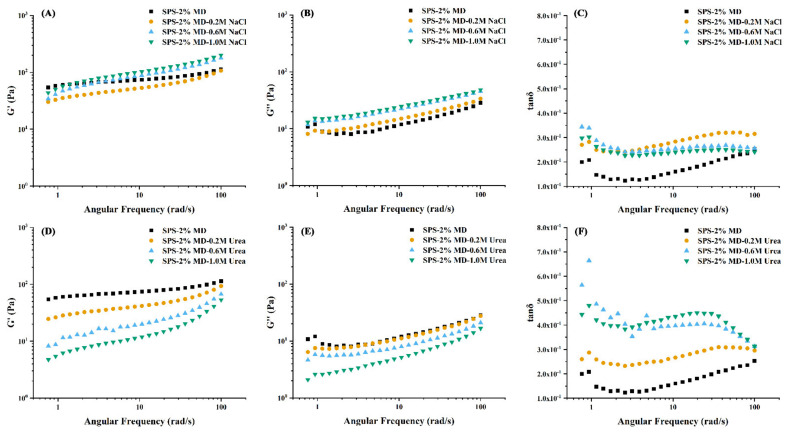
The storage (*G′*) and loss (*G″*) modulus, and $\tan\left(\delta \right)$ curves of sweet potato starch (SPS) gels with 2% (*w*/*w*) maltodextrin (SPS−2% MD) under different concentrations of NaCl (**A**–**C**) and urea (**D**–**F**).

The dynamic rheological properties of SPS-2% MD gels with the addition of sodium chloride (NaCl) and urea are illustrated in Figure 4. Storage modulus (*G′*) represents the elastic storage of energy, while loss modulus (*G″*) measures the dissipation of viscous energy. In this work (Figure 4A–D), no crossover point (*G′* = *G″*) was discovered and *G′* was always higher than *G″*, illustrating an elastic characteristic similar to a solid. With the enhancement of frequency, the relaxation time was cut down and a solid-like structure with more accessibility to an ordered network was gradually generated, thereby preserving the energy *G′* > *G″.* On the other hand, according to the structure of MD and SPS, electrostatic interaction and hydrogen bonds were proven to be the interaction force in prepared gels [21]. As shown in Figure 4A and D, the addition of urea and NaCl both caused a decrease in the *G′* value of SPS-2% MD gels, and the *G′* values of SPS-2% MD-urea and SPS-2% MD-NaCl gels were in the order of 0.2 M > 0.6 M > 1.0 M with the increasing angular frequency. Chen et al. (2014) also reported that pullulan can influence the structure of rice starch gels, thereby changing the dynamic modulus [35]. Herein, urea broke the hydrogen bonds between starch molecules and MD and among starch molecules. For NaCl, MD may react with sodium ions electrostatically, suppressing the interactions between MD and SPS, which further leads to the sliding and stretching of starch molecules with reduced elastic properties [42]. Comparatively, the effect of urea was stronger than that of NaCl. Additionally, the elastic and viscous properties were characterized by the ratio of *G″* to *G′* (tan δ). Generally, when *G′* is larger than *G″* (tan δ < 1), implying an elastic behavior, whereas there is a viscous behavior when *G′* is smaller than *G″*. In all cases, the tan(δ) was < 1 for all samples (Figure 4C and F), and the value increased with higher urea and NaCl content. The results indicated that the SPS-MD gels exhibited a solid-like behavior, and the addition of urea and NaCl led to less solid-like states of these pastes. Concurrently, urea demonstrated a stronger weakened effect than NaCl. In all, the hydrogen bond played a decisive role in MD-SPS forming a gel network structure. 

The relevant storage (*G′*) modulus parameters of the SPS-2% MD-urea and SPS-2% MD-NaCl gels are summarized in Table 4. The flow properties of all sample gels were well-fitted to the Power-Law equation (R^2^: 0.94–0.99). The value of K decreased with the addition of urea or NaCl, while the n value increased in SPS-2% MD gels (*p* < 0.05). Notably, this trend became more obvious when mixed with 1.0 M urea. The results suggested that urea and NaCl made gels sensitive to frequency, reducing the elastic properties of SPS-2% MD gels [43].

## 4. Conclusions

The performed study gave good insight into the effect of maltodextrin on the physicochemical, structural, and cooking characteristics of sweet potato starch noodles. In summary, the RVA results revealed that the addition of MD significantly decreased the peak and breakdown viscosity of SPS-MD gels. A low concentration of MD intensified the intra- and interchain interactions between starches and increased the crystallinity of SPS-MD complexes, which reduced the cooking loss and improved the chewiness. Additionally, sweet potato starch also formed a solid-like gel with MD, and the SPS-MD complex was mainly driven by hydrogen bonding as the gel strength decreased along with the increasing urea. However, higher concentrations of MD should be avoided as an excessive amount of free MD would weaken the starch gel structure, dissolve in boiling water, and finally lead to impaired cooking quality. These findings provide guiding information for the production of high-quality starch noodles, as well as the full utilization of sweet potato starches resources.

## Figures and Tables

**Table 1:** Cooking, texture properties, and FT-IR deconvolution results of sweet potato starch (SPS) noodles with various (0, 0.5, 1, 2, 3, and 5%, *w*/*w*) maltodextrin (MD) concentrations.

Sample	Cooking Behavior			Texture Parameters				
	Dry Matter Content (g/100g)	Swelling Index (%)	Cooking Loss (%)	Hardness (g)	Elasticity (mm)	Chewiness (g)	IR Ratio 1048/1020	IR Ratio 1020/996
SPS-0% MD	90.85 ± 1.47 ^a^	445.16 ± 20.60 ^b^	3.80 ± 0.50 ^b^	940.12 ± 37.25 ^d^	0.90 ± 0.02 ^ab^	728.17 ± 66.01 ^d^	0.878 ± 0.00 ^c^	1.115 ± 0.00 ^b^
SPS-0.5% MD	90.39 ± 2.22 ^a^	451.41 ± 17.56 ^ab^	1.47 ± 0.15 ^c^	1133.17 ± 84.08 ^c^	0.91 ± 0.03 ^ab^	894.05 ± 85.48 ^c^	0.869 ± 0.00 ^c^	1.162 ± 0.01 ^b^
SPS-1.0% MD	90.82 ± 2.03 ^a^	457.77 ± 23.83 ^ab^	2.14 ± 0.30 ^c^	1339.17 ± 52.85 ^b^	0.92 ± 0.02 ^a^	1079.70 ± 59.16 ^ab^	0.906 ± 0.00 ^ab^	1.061 ± 0.00 ^c^
SPS-2.0% MD	90.45 ± 1.74 ^a^	457.66 ± 11.16 ^ab^	2.16 ± 0.35 ^c^	1583.92 ± 89.09 ^a^	0.89 ± 0.03 ^ab^	1160.61 ± 92.52 ^a^	0.921 ± 0.00 ^a^	1.142 ± 0.01 ^b^
SPS-3.0% MD	90.78 ± 1.37 ^a^	460.04 ± 3.63 ^ab^	3.63 ± 0.28 ^b^	1511.83 ± 47.92 ^a^	0.89 ± 0.02 ^ab^	1132.53 ± 95.30 ^ab^	0.914 ± 0.01 ^a^	1.262 ± 0.03 ^a^
SPS-5.0% MD	90.8 ± 1.42 ^a^	474.74 ± 15.61 ^a^	4.69 ± 0.17 ^a^	1362.22 ± 117.86 ^b^	0.89 ± 0.01 ^b^	1047.57 ± 68.80 ^b^	0.889 ± 0.00 ^bc^	1.269 ± 0.01 ^a^

Different superscript letters between columns indicate significant differences (*p* < 0.05).

**Table 2:** Pasting property of sweet potato starch (SPS) with various (0, 0.5, 1, 2, 3, and 5%, *w/w*) maltodextrin (MD) concentrations.

Sample	Peak Visc (cps)	Through Visc (cps)	Breakdown (cps)	Final Visc (cps)	Setback (cps)
SPS-0% MD	1137.8 ± 11.75 ^a^	924.0 ± 5.90 ^a^	214.2 ± 7.47 ^ab^	1233.8 ± 8.61 ^a^	310.6 ± 7.91 ^a^
SPS-0.5% MD	1122.8 ± 4.62 ^b^	914.2 ± 6.27 ^b^	206.4 ± 7.00 ^bc^	1223.4 ± 12.08 ^ab^	309.4 ± 9.65 ^a^
SPS-1.0% MD	1108.4 ± 5.82 ^c^	910.2 ± 5.19 ^b^	197.8 ± 9.56 ^c^	1210.2 ± 10.57 ^b^	299.8 ± 5.74 ^ab^
SPS-2.0% MD	1080.4 ± 9.83 ^d^	881.6 ± 9.65 ^c^	197.6 ± 5.57 ^c^	1179.0 ± 12.47 ^c^	295.4 ± 10.09 ^ab^
SPS-3.0% MD	1056.6 ± 3.44 ^e^	859.0 ± 6.23 ^d^	197.2 ± 5.04 ^c^	1143.8 ± 18.70 ^d^	285.2 ± 13.42 ^b^
SPS-5.0% MD	1001.6 ± 3.77 ^f^	782.6 ± 3.61 ^e^	218.4 ± 5.92 ^a^	1038.2 ± 14.43 ^e^	255.8 ± 10.91 ^c^

Different superscript letters between columns indicate significant differences (*p* < 0.05).

**Table 3:** Gelatinization parameters of sweet potato starch (SPS) noodles with various (0, 0.5, 1, 2, 3, and 5%, w/w) maltodextrin (MD) concentrations.

Sample	ΔH (J/g)	To (°C)	Tp (°C)	Tc (°C)
SPS-0% MD	2.55 ± 0.6 ^b^	57.96 ± 0.54 ^ab^	64.31 ± 0.14 ^a^	70.62 ± 0.89 ^a^
SPS-0.5% MD	6.08 ± 0.29 ^a^	58.27 ± 0.02 ^ab^	63.77 ± 0.07 ^a^	69.47 ± 0.33 ^ab^
SPS-1.0% MD	6.01 ± 0.54 ^a^	55.36 ± 1.54 ^b^	61.78 ± 0.35 ^b^	67.61 ± 0.48 ^c^
SPS-2.0% MD	6.31 ± 0.11 ^a^	56.17 ± 0.28 ^ab^	61.53 ± 0.24 ^b^	67.97 ± 0.4 ^bc^
SPS-3.0% MD	6.23 ± 0.37 ^a^	56.68 ± 1.01 ^ab^	60.95 ± 1.69 ^b^	67.55 ± 1.49 ^c^
SPS-5.0% MD	5.64 ± 0.52 ^a^	56.94 ± 0.28 ^ab^	60.42 ± 0.26 ^b^	64.56 ± 0.43 ^d^

ΔH, gelatinization enthalpy; To, initial temperature; Tp, peak temperature; Tc, end temperature. Different superscript letters between columns indicate significant differences (*p* < 0.05).

**Table 4:** Dynamic storage (*G′*) modulus parameters of sweet potato starch (SPS) gels with 2% (*w*/*w*) maltodextrin (SPS-2% MD) under different concentrations of urea and NaCl.

Sample	*K* (Pa·s*^n^*)	*n*	R^2^
SPS-2% MD	56.79 ± 0.77 ^a^	0.12 ± 0 ^e^	0.96
SPS-2% MD-0.2M NaCl	31.60 ± 1.92 ^c^	0.23 ± 0.02 ^d^	0.98
SPS-2% MD-0.6M NaCl	45.98 ± 1.60 ^b^	0.29 ± 0 ^c^	0.98
SPS-2% MD-1.0M NaCl	55.03 ± 0.42 ^a^	0.27 ± 0 ^c^	0.99
SPS-2% MD-0.2M Urea	27.25 ± 1.69 ^d^	0.22 ± 0.01 ^d^	0.95
SPS-2% MD-0.6M Urea	10.15 ± 0.82 ^e^	0.34 ± 0.02 ^b^	0.95
SPS-2% MD-1.0M Urea	5.18 ± 0.06 ^f^	0.41 ± 0.01 ^a^	0.94

Different superscript letters between columns indicate significant differences (*p* < 0.05).

## Data Availability

Data available on request due to restrictions, e.g., privacy or ethical.
